# Reader reception of translation strategies for Li Bai’s “Yu Jie Yuan”: Empirical evidence from a mixed-methods study

**DOI:** 10.1371/journal.pone.0348360

**Published:** 2026-05-05

**Authors:** Hongjing Chang, Jing Zhao, Yuhao Sun, Huan Ding

**Affiliations:** 1 School of Foreign Languages, Quzhou University, Quzhou, China; 2 School of Languages, Literacies and Translation, Universiti Sains Malaysia(USM), Penang, Malaysia; 3 Anhui Huaren Law Firm, Hefe, China; Bahir Dar University, ETHIOPIA

## Abstract

Classical Chinese poetry faces challenges in cross-cultural communication due to differences in rhythm, imagery, and poetic traditions. Although various translation strategies have been proposed, empirical research on how target readers evaluate them remains limited. This study adopted a mixed-methods approach to examine four English translations of Li Bai’s “Yu Jie Yuan”: prose translation, free verse translation, rhymed translation, and literal translation. A total of 403 American audiences were recruited through the Prolific platform and completed an online questionnaire on Qualtrics. The adapted Reader Response Questionnaire (RRQ) covered five dimensions: “content, theme, affective involvement, character behavior, and typology”. Quantitative data were analyzed using t-tests and analysis of variance, while open-ended responses were subjected to qualitative thematic analysis. In addition, four English translations of Li Bai’s “Yu Jie Yuan” were examined through textual analysis. The results showed that among the four strategies compared, rhymed translation was more popular with readers, indicating its potential advantage in balancing meaning and aesthetics. This study provided empirical support for the role of translation strategies in cross-cultural communication. However, since the sample was limited to American audiences, future research should expand to other cultural groups to verify the generalizability of the conclusion.

## 1. Introduction

Classical Chinese poetry (CCP) represents one of the most influential poetic traditions globally, celebrated for its dense imagery, philosophical depth, and cultural specificity. As part of the broader field of intercultural communication, the translation of CCP is conceptualized not merely as linguistic conversion but as a mediating practice that negotiates aesthetic norms, cultural values, and readers’ interpretive expectations across languages, foregrounding the role of translation in cultural exchange and communication [1,2]. Despite its cultural significance, the translation of CCP has long been a site of controversy with concerns over whether and how verse should be rendered into verse, particularly regarding the retention of formal features such as rhyme, meter, and lineation. From a reception-oriented perspective, these strategies can also be understood as implicit hypotheses about target readers’ reading preferences with recent scholarship emphasizing systematic evaluation frameworks, the interplay between translation strategies and target readers’ cultural and aesthetic expectations, as well as reader immersion in poetic translation [3–5].

A seminal attempt to systematize poetry translation strategies is proposed by André Lefevere, who identifies seven strategies, including phonemic translation, literal translation, metrical translation, poetry into prose, rhymed translation, blank verse translation and interpretation, and argues that no single strategy can fully reproduce all features of the source poem [6]. Although Lefevere’s typology is developed primarily in relation to Indo-European languages, it has exerted lasting influence on Chinese scholars’ understanding of CCP translation. In terms of the English translation of CCP, there are mainly three translation strategies —“rhymed translation, free verse translation and prose translation” [7]. Herbert Allen Giles is a representative advocate of rhymed translation in early English renderings of CCP. Grounded in the belief that Chinese poems are originally lyric compositions intended for musical performance, Giles argues that rhyme is essential for preserving their poetic nature in English. Drawing on the English lyrical tradition, he echoes A.C. Swinburne’s view that rhyme constitutes a defining feature of English lyric poetry. This approach aligned CCP translation with the dominant poetics of Victorian England and shaped the first major wave of CCP translation and study in the West [8]. The most influential Chinese successor of Hebert Giles’ rhymed translation is Xu Yuanchong (2016), who emphasizes the recreation of poetic music and structural elegance, guided by his Three Beauties theory, advocating beauty in sense, sound, and form.

Free verse translation preserves lineation and imagistic intensity without adhering to strict rhyme schemes. After the 1920s, the Western society, economy and culture changed dramatically, and the rhymed translation was increasingly criticized by the free verse translators represented by Pound and Waley because of its deviation from the mainstream poetics. Ezra Pound’s *Cathay* exemplifies this approach, foregrounding direct presentation of images and paratactic rhythm in English. To abandon traditional rhyme, Pound the poet broke the pentameter and self-consciously resorted to French symbolists and deployed strophic verse-forms with line-breaks and mid-line caesurae. By dint of line-breaks, each poetic line could be a “musical phrase” — a self-contained rhythmic and grammatical unit so that the images could be highlighted. Pound’s publishing of *Cathay* in 1915, brought him high fame as an innovative poet. Two years later, Waley’s *A Hundredand Seventy Chinese Poems* appeared, making him another rising star translating CCP in free verse. Arthur Waley combining English free verse with Chinese versification, developed a new form of free verse in translating CCP—“sprung rhythm” method. According to Waley (1918), it is impossible to reproduce the effect of rhyme in English like those of CCP in which the same or similar rhyme might appear throughout a whole poem. Rhymed translation necessarily “injures either the vigor of one’s language or the literalness of one’s version” [9].

In prose translation, the original poem is converted into a prose without lineation or rhymes and the inner rhythm inside the sentence is often adopted to reflect the rhyming beauty of the original poem. There are mainly three noticeable translators translating CCP into English prose—David Hawkes and William Hung and Weng Xianliang [10]. David Hawkes adopts different translation strategies when translating different literature. He uses rhymed translation in translating the poems in *A Dream of Red Mansions* and *Ch’u Tz’u: The Songs of The South*, *An Ancient Chinese Anthology* while adopting prose form in translation Du Fu’s (also known as Tu Fu) poems. Zhu [8 believed that there were two reasons for Hawks’ adopting prose form in *A Litter Primer of Tu Fu*: First, Hawks’ translated work *A Litter Primer of Tu Fu* aims to introduce Du Fu’s poems to Western readers. The acceptability of translated poems is his primary concern. Second, Du Fu’s poems have profound cultural deposits with refined and rigorous forms, which are difficult to preserve in refined poetic forms like rhymed form or free verse.

William Hung (1893–1980), a Chinese historian and sinologist, is renowned for his biography of Du Fu. William Hung is not a professional translator. He just translates part of Du Fu’s poems in the process of studying Du Fu, making his translation streaked with the nature of academic research. William Hung’s prose translation deserves praise in terms of faithfulness and readability [11]. However, although William Hung’s translations are commendable in some respects, their lack of poetic flavor is also obvious [8]. Weng Xianliang is the most prominent Chinese translator translating CCP into English prose. His artistic pursuit is to highlight the ideorealm of the original texts, which gives rise to the lasting appeal of his translations. Weng’s prose-like translations can mainly be found in his collection *An English Translation of Ancient Poems* (1985) in which 117 classical Chinese poems were selected, including 10 poems by Li Bai. In the preface to the book, Weng Xianliang maintains that “Poetry can express emotion; yet emotion is not necessarily expressed by poetry. The poet’s emotion is conceived in the image; the poet’s thought is consonant with the poem’s sound. The difficulty in translating poetry lies in reproducing the image and modifying the sound. Reproducing images cannot deviate from the poet’s original intention...Modifying the sound should not be bound by traditional poetic forms. It doesn’t matter if the poem rhymes or not, nor is it a big deal if it breaks the line or not...” [12]. Weng Xianliang’s prose translation is based on his understanding of the essence of poetry and translation, which has unique poetic brilliance. His unique translation artistic thoughts, like a spring of fresh water, inject new vitality into the translation of ancient Chinese poetry in the modern context.

Apart from the previous translation strategies, word-for-word translation is also necessary. As Nida [13] suggests, some readers need strict word-for-word translations because they want to understand the formal characteristics of the source text and avoid over-interpretation by the translator himself. The Argentine novelist, poet, and translator Jorge Luis Borges [14] argues that literal translation has theological origins and readers prefer word-for-word translations because they can achieve the effect of beauty of singularity and strangeness, although word-for-word translations may still be a crime in the eyes of some translators in the past. These contrasting positions reveal that disagreements over translation strategies are not merely technical or stylistic, but fundamentally reader-oriented, as each strategy presupposes a different model of the target reader’s aesthetic competence and expectations.

## 2. Literature review on readers’ reception in translation

Reception, as defined by Karas [15], concerns how a target culture responds to translated texts in terms of its dissemination, and its contents. Building on this notion, Chesterman [16] conceptualizes translation reception through reactions, responses, and repercussions, while Gambier [17] further refines these categories by emphasizing readers’ preferences and expectations as key indicators of reception. Subsequent empirical studies, particularly in reader-oriented translation research, have demonstrated that readers’ emotional responses, attitudes, evaluations, and expectations constitute measurable dimensions of reception, and that questionnaires and interviews offer effective methodological tools for capturing such data (Suojanen et al. [18]). However, although reception studies increasingly foreground readers as active evaluators, much of the existing research treats reception as a relatively autonomous phenomenon, insufficiently linked to specific textual or translational variables. Studies such as Khoshsaligheh et al [19], which investigate readers’ preferences for translated novels, implicitly assume that translation choices shape reader expectations, yet they rarely isolate or systematically compare concrete translation strategies as explanatory factors.

This limitation is particularly evident in studies on the reception of English translations of Chinese literature. Due to the heterogeneity and fluidity of Western readerships, empirical research remains scarce, with only “a handful of studies” addressing this area [20]. Existing research has largely relied on indirect indicators of reception, such as literary awards, library holdings, media visibility, sales figures, and reader familiarity (Bao [21]), reader surveys and online reviews (Ji [22]), archival materials such as letters and book reviews (Zheng [23]), or interviews combined with publication histories and critical reception (Wang [24]). While these studies have established the importance of reader evaluation in assessing the overseas reception of Chinese literature, they tend to focus on macro-level outcomes rather than on how specific translation strategies condition readers’ responses at the textual level. Consequently, despite growing recognition of the centrality of reader reception, there remains a lack of empirical research that directly links readers’ reception to identifiable translation strategies —a gap that becomes particularly pronounced in the case of poetry translation.

In the realm of poetry translation, the strategies employed when translating CCP are crucial in bridging the gap between cultures. At the end of the 19th century, the rhymed translation representative Herbert Giles translated classical Chinese poems in a rhymed antiquarian style, with great neatness and elegance, which was highly praised by the English critic Lytton Strachey, who described his translations as “beautiful, novel, and full of charm” [25]. Rhymed translation was increasingly criticized by the free verse translators represented by Pound and Waley after the 1920s because of its deviation from the mainstream poetics. In the 21st century, translating ancient Chinese poems into unrhymed free verse in modern English is a common method adopted by contemporary translators [26]. According to Ma [27], “Xu Yuanchong’s translating ancient Chinese poems with rhymed English translation in modern times is undoubtedly a kind of epochal dislocation since he opted for Giles’ rhymed translation while abandoning Waley’s free verse translation”. Zhang [28] also advocated that in the English translation of ancient Chinese poems, only free verse translation can match the trend of contemporary English poetry, win the Western readers, and ultimately contribute to the communication of ancient Chinese poetry to the Western world. There are also scholars who hold a different opinion. The British sinologist Arthur Cooper [29] commented: “Most of today’s ‘free verse’ is closer to the Chinese ‘fu’ than to ‘ci’ or ‘poem’, which is an inherent error in trying to translate ancient Chinese metrical poetry into free verse or prose. According to Li and Yan [30], “We should not assume that just because some Western sinologists don’t use rhymed translation, it is wrong for Chinese translators to use it, and it is even more wrong for some Western sinologists to ask us not to rhyme in translating ancient Chinese poems”.

Responding to both the empirical gap in reception studies and the theoretical controversy over translation strategies of CCP, this study conceptualized reader reception and translation strategy as interrelated dimensions of the same translational phenomenon, offering a more integrated account of how classical Chinese poetry is received in the English-speaking world. The study investigated target readers’ preferences for four translation strategies—prose translation, free-verse translation, rhymed translation, and word-for-word translation through a mixed-methods approach combining a questionnaire survey and qualitative textual analysis of four English versions of Li Bai’s “Yu Jie Yuan”. In doing so, it contributed to a more integrated, reader-oriented understanding of poetry translation and to inform the optimization of CCP translation for cross-cultural communication. Specifically, the study addressed the following questions: 1) what are target readers’ evaluations on four translation strategies, i.e., prose translation, free verse translation, rhymed translation and word-for-word translation of classical Chinese poetry? 2) What translation strategy should be adopted in translating classical Chinese poetry?

## 3. Research methodology

### 3.1. Research corpus

The original poem “Yu Jie Yuan” (玉阶怨, literally meaning Jade Steps Grievance,), is a pentasyllabic four-line regulated verse (i.e., five-character-per-line wuyan lüshi). It is chosen as corpus in the questionnaire because it represents classical Chinese poetry’s essential qualities of conveying “images” with “vivid presentation” and “marked reticence”, elsewhere referred to as “obscurity” [31]. It is a quintessential example of palace grievance poetry (gōng yuàn shī 宫怨诗), a popular form of yue-fu poetry in the Han and Tang Dynasties, and serves mainly for the description of women who complain of their dissatisfaction with the luxurious but lonely life. The whole poem is highly implicative and reserved in expressing thoughts and feelings. Li Bai expressed the loneliness of the protagonist’s life by describing the details of her life and psychological dynamics. There is deep resentment expressed in the protagonist; albeit no word of “complaint” written in the poem. This poem, conveying deep and profound beauty, is a treasure of classical Chinese poetry.

The four translations selected are made by renowned representative translators in different translation strategies of CCP. Version 1 “The Night Is Well On” is translated by Weng Xianliang, a famous translator in China, whose translation book *Ancient Chinese Poems in English* [32] established his position as “a representative of the prose translation school of poetry translation in” China [33]. Version 2 “The Jewel Stairs’ Grievance” is translated by the modernist poet Ezra Pound, who is a leader of the New Poetry Movement in America, and his translation collection *Cathay* inspired numerous translators to abandon fixed metrical pattern and adopt free verse in translating classical Chinese poetry [34]. Version 3 “Waiting in Vain on Marble Steps” is translated by Xu Yuanchong [35], who, as the most famous contemporary translator in China, inherits Herbert Giles’ rhymed translation and is considered by Gu Zhengkun as “the translator who has made the greatest contribution to translating Tang poems into English in the world” [36].

### 3.2. Research instrument

In this research, Reader Response Questionnaire (RRQ) was set up based on Zaharias and Mertz’s [37] modification of Alan Purves’ Reader Preference Measure (RPM) [38]. RRQ contains Part One and Part Two. Part One is composed of 4 fact-finding background questions on demographic information to elicit personal data, focusing on the participants’ age, gender, education, occupation. Part Two involves comparison of 4 different translation strategies of Li Bai’s poem “Yu Jie Yuan” with 5 fixed structured questions mainly reflected in five elements chosen to be the most valuable by the participants in Zaharias and Mertz’s research— “content”, “theme”, “affective involvement”, “character behavior” and “typology” [37], which are answered with 5-point Likert scale along a continuum of “very poor”, “poor”, “average”, “good”, “excellent”. It is worth noting that, in a broader literary context, “typology” involves identifying content, themes, characters, events, etc. in a literary work that serve as types in general. Therefore, the fixed structured question reflected in “typology” in RRQ can be the overall evaluation of the translation. The five questions in RRQ corresponding to the five elements are as follows.

1) To what extent do you think the translation reflects the content of the original poem through its form, diction and sound?
2) To what extent do you think the translation reflects the hidden motif of the original poem through its imagery?
3) To what extent do you think the translation succeeds in evoking your emotions and getting you involved in the poetic atmosphere?
4) To what extent do you think the translation explains the character behavior in the original poem?
5) To what extent do you like the translation in general?

It is worth noting that, the nationality and name of the translators are not be mentioned in the questionnaire so as not to influence their evaluation. The original poem is provided with notes to facilitate participants’ reading. Ideally, though, the quality assessment of the translation should be carried out by a proficient bilingual based on comparison between ST and TT, in which case, both “adequacy” and “acceptability” of the translation could be taken into consideration [39]. But in practice, most target readers do not understand ST, and their evaluation of translation is based on reading monolingual translation. For them, comparison of multiple translations is a feasible means to judge the quality of translation. Thus, readers could garner different reading experience brought to them by reading different translations and comparing the effects of different translations based on its “content”, “theme”, “affective involvement”, “character behavior” and “typology”. In order to further examine readers’ responses on the four translations, the researcher set up an open-ended question, namely, “Please make some comments on your most favorite version” for more detailed and systematic analysis of the results.

### 3.3. Research model

The research adopted both product-oriented and participant-oriented research model [40] with the focus of study on not only translated texts but also on the participants involved in the research process. The product-oriented research was carried out through textual analysis. Textual research “focuses on texts themselves as linguistic data” and “looks at the relations between translations, their source texts, and parallel non-translated texts in the target language” [41]. By means of textual analysis, the researcher identified the features of four translation strategies of Li Bai’s poem “Yu Jie Yuan”. The participant-oriented research was carried out through questionnaire survey with both closed and open questions to investigate the target readers’ evaluation on four English translation strategies of “Yu Jie Yuan”. Open-ended responses were subjected to qualitative thematic analysis. Participants’ comments were hand-coded and analyzed. The researcher statistically analyzed subjects’ comments regarding the five elements in the RRQ: “content,” “theme,” “affective involvement,” “character behavior,” and “typology.” A deductive coding approach, which is concept-driven or theory-driven, was employed: the researcher began with a predefined set of codes corresponding to the five elements and assigned them to the open-ended comments. Comments that involved more than one element were coded multiple times. After the initial coding, a partner coder (i.e., the corresponding author), familiar with the study, verified the codes to enhance the credibility and validity of the qualitative data.

### 3.4. Theoretical framework

This study adopts Xu Yuanchong’s [42] theory of literary translation as an analytical and evaluative framework for examining the relationship between translation strategies and reader reception of CCP. Mr. Xu Yuanchong’s translation theory is considered to be “the most brilliant summary of translation theory by Chinese scholars in the last hundred years” [36]. The study mainly draws on the core concepts—Three Beauties Theory (san mei lun), Three-izations Theory (san hua lun), and the teleological and epistemological principles—to guide questionnaire construction, and textual analysis.

#### 3.4.1. Three beauties theory: Ontology of poetry translation.

Xu’s Three Beauties Theory identifies three dimensions of aesthetic value in poetry translation: beauty in sense, sound, and form [42]. Beauty in sense concerns the accurate and vivid transmission of the original poem’s meaning, imagery, and artistic conception. Beauty in sound refers to the rhythm, rhyme, and phonetic resonance of the translated text, contributing to readers’ aesthetic and emotional engagement. Beauty in form addresses the structural correspondence between source and target texts, including line length and overall layout. In this study, these dimensions serve as evaluative criteria for both questionnaire design and textual analysis, allowing researchers to examine how different translation strategies foreground or compromise each aesthetic aspect.

#### 3.4.2. Three-izations theory: Methodology of translation.

Xu’s Three-izations Theory—generalization (qian hua), particularization (shen hua), and equalization (deng hua)—provides methodological guidance for analyzing translational decisions [42]. From a broader translation-theoretical perspective, the Three-izations theory has been constantly interpreted in dialogue with established translation-theoretical techniques for poetry translation [43–45]. Generalization resonates with simplification-oriented strategies that aim to reduce cultural density and cognitive load for target readers, particularly in the translation of culturally embedded poetic imagery. Particularization, by contrast, aligns with explicitation and enrichment strategies, whereby translators add interpretive detail to render compressed meanings and aesthetic nuances accessible in the target language. Equalization (denghua) largely corresponds to establish equivalence in translation theory. These techniques inform textual analysis of Li Bai’s “Yu Jie Yuan”, enabling the identification of how each translation strategy applies Xu’s procedural logic.

#### 3.4.3. Teleology and epistemology: From understandability to aesthetic delight.

Xu [42] emphasizes that literary translation must fulfill three teleological levels: understandability, enjoyability, and delectability. Understandability ensures that readers can comprehend the poem’s content, represented in the questionnaire via content and character behavior items. Enjoyability captures readers’ emotional engagement and aesthetic response, reflected in affective involvement items. Delectability represents the highest achievement, integrating sense, sound, and form to maximize aesthetic pleasure, assessed via typology/overall evaluation. Epistemologically, Xu’s notion of “creation of the best as in rivalry” frames literary translation as a creative negotiation between cultures. This perspective justifies the comparative analysis of multiple translation strategies and supports the study’s focus on how actual target readers perceive and evaluate these strategies.

#### 3.4.4. Operationalization of Xu Yuanchong’s theory of literary translation.

The theoretical framework directly informed the study’s Reader Response Questionnaire (RRQ). The five evaluative dimensions—content, theme, affective involvement, character behavior, and typology—are explicitly mapped to Xu’s theoretical concepts. Content and theme correspond to beauty in sense. Affective involvement aligns with beauty in sound and the teleological criterion of enjoyability. Character behavior reflects Xu’s equalization and particularization, supporting comprehension and interpretive clarity. Typology serves as an overall evaluative category, integrating the Three Beauties and reflecting holistic aesthetic success. Through this alignment, the study ensures that the questionnaire and textual analysis are conceptually grounded, allowing for an empirical examination of how Xu Yuanchong’s aesthetic principles are realized and perceived in the target language.

### 3.5. Sampling

Convenience sampling was employed in this study, with participants recruited through the online platform Prolific to complete the electronic questionnaire on Qualtrics. Prolific is an online platform that links researchers with subjects for academic research, which offers ethical research procedures, transparent pricing and high-quality data, etc. Compared with traditional internet-based sampling, Prolific can provide more samples with better geographical and occupational diversity (the researcher can screen and qualify samples by specifying their gender, age, education, origin region, etc.). Douglas, et al. maintained that compared to MTurk (another commonly used online platform) or an undergraduate student sample (i.e., SONA), participants on Prolific and CloudResearch were more likely to pass various attention checks, provide meaningful answers, follow instructions, remember previously presented information, have a unique IP address and geolocation, and work slowly enough to be able to read all the items; thus, Prolific and CloudResearch provided the highest quality data for the lowest cost [46]. As Palan & Schitter claimed, Prolific not only has detailed rules regarding the treatment of subjects on the platform, but also has a user-friendly interface, and features a superset of MTurk’s functions [47]. Up till now, Prolific has registered thousands of researchers, many of whom have successfully utilized it as a subject pool in their respective fields, such as food science [48], psychology [49], or economics [50].

Reader reception of poetry is inevitably mediated by culturally specific literary conventions, educational backgrounds, and aesthetic norms. Consequently, cross-cultural reception studies should be understood not as the search for universal reader responses, but as contextually grounded inquiries into how meaning is negotiated within particular cultural settings. This sampling choice was intended to situate the reception of classical Chinese poetry within a context shaped by American literary traditions, translation norms, and reading habits. By limiting participants to readers based in the United States, the study examined how CCP is interpreted and evaluated within a particular Western cultural milieu, thereby allowing for a more controlled and context-sensitive analysis of cross-cultural reception. This context-bound approach necessarily entailed limitations in terms of generalizability; however, it also enabled a focused investigation into the interaction between translated poetic texts and American readers’ culturally conditioned expectations, aesthetic preferences, and interpretive frameworks. Adam’s Table is employed to determine the sample size since it is more comprehensive and rigorous which determined the optimum sample size at three commonly used confidence levels: 90%, 95% or 99% with a margin of error of 3% for continuous data and 5% for categorical data, as can be shown from Table 1 [51]. Therefore, in reference to Adam’s Table (see Table 1), this study designed quantitative sampling with about 400 for continuous data in 5-scale Likert questionnaire.

**Table 1:** Adam’s (2020) Table for Determining Sample Size for a Given Population Size for Continuous Data.

Population size	Sample Size					
	Categorical data (margin of error = .05), ρ= 2			Continuous data (margin of error = .03), ρ = 4		
	90% confidence Level t = 1.645	95% confidence Level t = 1.96	99% confidence Level t = 2.58	90% confidence Level t = 1.645	95% confidence Level t = 1.96	99% confidence Level t = 2.58
450	169	208	269	133	168	229
500	176	218	286	137	174	241
600	187	235	316	144	185	262
700	196	249	342	149	194	279
800	203	260	364	153	201	293
900	209	270	383	156	206	306
1000	213	278	400	159	211	317
1200	221	292	429	163	219	334
1500	230	306	462	167	227	354
2000	239	323	500	172	236	376
3000	249	341	545	177	245	401
5000	257	357	588	182	254	424
8000	262	367	615	184	259	437
10000	264	370	625	185	260	442
20000	267	377	645	187	264	452
50000	270	382	657	188	266	459
100000	270	383	662	188	267	461
150000	271	384	663	188	267	461
200000	271	384	664	188	267	462
>1000000	271	385	666	188	267	463

### 3.6. Research procedures

The researcher firstly created an online questionnaire link through Qualtrics platform and set up a 5-digit random code at the end of the questionnaire items, which was used to screen and identify the qualified samples in Prolific to ensure the authenticity and validity of the sample data. Due to the inherent incentive mechanism and highly remunerating payment on the Prolific platform, the questionnaire samples generally responded quickly and showed high motivation and conscientiousness in completing the questionnaire. After the samples completed the questionnaire, the researcher would pay them promptly.

The researcher was able to screen the samples based on their questionnaire response time and content quality without having to forcefully approve any questionnaire results they submitted. Therefore, before distributing the questionnaires, the researcher firstly restricted sample size, the location and nationality of the samples and then excluded questionnaires with omissions or questionnaire response time of less than 3 minutes in order to clarify the scope of the samples, optimize the sample feedback, and improve the quality of the questionnaire data.

### 3.7. Ethical statement

This ethical review of the study was approved by the School of Foreign Languages at Chuzhou University (No. C2SC2025−022). The study did not involve minors or other vulnerable groups, and only collected anonymous questionnaire data, with extremely low risk. A total of 403 adult volunteers were recruited via the Prolific academic research platform. Prior to participation, each respondent viewed an informed consent statement and indicated consent by clicking “Agree” on the Qualtrics survey. Participation was voluntary, anonymous, and non-invasive, and samples could withdraw at any point without penalty.

## 4. Results

### 4.1. Reliability and validity

The demographic characteristics of the 403 samples who participated in the RRQ for classical Chinese poetry in this study are shown in the table below.

As Table 2 indicates, samples RRQ are also mostly young and well-educated, aged between 17–59 (92.6%) with more than half of samples (57.8%) obtaining at least bachelor’s degrees. In terms of gender, the two groups are not far apart. There is a nearly even split between the number of men (42.9%) and women (57.1%). All of the samples are from the U.S. and are located in various states of the country, such as Missouri, Massachusetts, Michigan, California, etc. They also have diverse occupations (manager, employee, QC analysts, financial manager, flight attendant, Japanese tutor and school principal, etc.). The Reliability of RRQ on 4 versions among 403 samples is measured through Cronbach α and split-half reliability. As can be shown on the Table 3, both of the numerical values are above 0.9, indicating excellent reliability of the instrument.

**Table 2:** Demographic Characteristics of TC Audience in RRQ.

Basic Information of Samples		Number	Percentage (%)
Age	17-29	128	31.8
	30-59	245	60.8
	60-	30	7.4
Gender	Male	173	42.9
	Female	230	57.1
	Primary and middle school qualifications	2	0.5
	High school qualification	107	26.6
	U.S. career and technical qualifications	61	15.1
	Bachelor’s degree	172	42.7
	Master’s degree	48	11.9
	Doctorate degree	13	3.2

**Table 3:** Reliability of RRQ Among 403 Samples.

RRQ on TCCP	Items	Cronbach α	Split-half reliability
Version 1	5	0. 905	0.906
Version 2	5	0.928	0.928
Version 3	5	0.928	0.928
Version 4	5	0.951	0.951

Communalities variance analysis is a commonly used method to assess the structural validity of a questionnaire. The communalities variance represents the sum of the variance of each observed variable (question or measurement item) with respect to all the communal factors (underlying constructs or themes), and it can be used to assess the extent to which each observed variable is explained by the communal factors. Initial suitability of data for factor analysis is employed before applying communalities variance analysis. As can be shown from the Table 4, KMO values of the 4 versions are 0.889, 0.901, 0.904 and 0.912 respectively (>0.8); all the P values of 4 versions from Bartlett’s Test of Sphericity are lower than 0.05. Therefore, it is reasonable to say that the data from RRQ is very suitable for communalities variance analysis.

**Table 4:** Initial Suitability of Data for Factor Analysis in RRQ.

KMO and Bartlett’s test of Sphericity		Version 1	Version 2	Version 3	Version 4
KMO values		0.889	0.901	0.904	0.912
Bartlett’s test of Sphericity	Approx. Chi-Square	1274.915	1568.386	1522.751	2004.508
	degree of freedom	10	10	10	10
	Significance level	0.000	0.000	0.000	0.000

To analyze the structure validity of the questionnaire based on the communality variance provided, the initial and extracted communalities need to be evaluated for each item. The value of the initial common factor variance is usually set to 1 because the initial common factor variance is calculated assuming that the variance of each observed variable can be fully explained by all extracted principal components. The extracted communalities (also known as extracted common factor variance) are typically values between 0 and 1. These values represent the proportion of each observed variable’s variance that is explained by the extracted principal components or factors. Higher extracted communality values closer to 1 suggest better structure validity, as they indicate that a larger proportion of each observed variable’s variance is explained by the underlying factors.

To investigate whether the 5 items in each version of RRQ have structure validity, communalities variance analysis is employed. As shown in Table 5, all the extracted communalities are closer to 1, showing consistency across 4 versions of the questionnaire, with some slight variations in the communalities. Thus, in general, the data on RRQ demonstrates reasonable to strong structure validity across different items (“content”, “theme”, “affective involvement”, “character behavior” and “typology”) and versions. Particularly, the “typology” item consistently shows higher extracted communalities across all versions, indicating stronger structure validity.

**Table 5:** Communalities Variance Analysis of Data in RRQ on TCCP.

Communalities Variance		
Items	Initial Communalities	Extracted Communalities
Version 1_content	1.000	0.765
Version 1_theme	1.000	0.677
Version 1_affective involvement	1.000	0.712
Version 1_character behavior	1.000	0.660
Version 1_typology	1.000	0.834
Version 2_content	1.000	0.756
Version 2_theme	1.000	0.766
Version 2_affective involvement	1.000	0.795
Version 2_character behavior	1.000	0.712
Version 2_typology	1.000	0.863
Version 3_content	1.000	0.761
Version 3_theme	1.000	0.763
Version 3_affective involvement	1.000	0.794
Version 3_character behavior	1.000	0.728
Version 3_typology	1.000	0.835
Version 4_content	1.000	0.810
Version 4_theme	1.000	0.842
Version 4_affective involvement	1.000	0.834
Version 4_character behavior	1.000	0.822
Version 4_typology	1.000	0.877

### 4.2. Samples’ evaluations on four translations

Since the items in the 4 translations are continuous numerical variables, descriptive statistical analysis and one-sample T-test are used to examine samples’ evaluations on the 4 translations.

As shown in Table 6, the P values of the one-sample t-tests for the four translation strategies and the overall translations are all below 0.05, indicating statistically significant and reliable results. The mean values of prose translation, free verse translation, rhymed translation, and overall translations are all significantly higher than the neutral value of 3, suggesting a generally positive reception of these strategies among the samples. By contrast, the word-for-word translation strategy receives a mean value below 3, indicating relatively low reader satisfaction. Among the four English translations examined, Xu Yuanchong’s rhymed version “Waiting in Vain on Marble Steps” (Version 3) achieves the highest mean score (3.71), followed by Weng Xianliang’s prose translation “The Night Is Well On” (Version 1) with a mean value of 3.44. Notably, both highly rated versions are produced by native Chinese translators, suggesting that culturally informed aesthetic mediation may play a role in shaping reader reception.

**Table 6:** Samples’ Evaluations on 4 Translations in RRQ Based on Descriptive Statistical Analysis and One-Sample T Test.

RRQ on TCCP	No.	Minimum value	Maximum value	Mean value± standard deviation	Test value	T value	P value
Prose translation	403	1.00	5.00	3.44 ± 0.78	3	11.216	<.001
Free verse translation	403	1.00	5.00	3.16 ± 0.84	3	3.735	<.001
Rhymed translation	403	1.40	5.00	3.71 ± 0.78	3	18.355	<.001
Word-for-word translation	403	1.00	5.00	2.63 ± 0.99	3	−7.539	<.001
Overall translations	403	1.60	5.00	3.23 ± 0.55	3	8.569	<.001
No. of valid cases	403						

As shown in Table 7, 181 samples (44.9%) explicitly identified the rhymed version as their favorite, followed by prose translations (29.5%) and free-verse translations (15.6%). The quantitative preference for rhymed translation is further corroborated by the open-ended responses. Rather than serving merely as illustrative comments, these qualitative responses provide interpretive evidence for understanding why rhyme resonates with readers. Many samples associated rhyme with “musicality,” “memorability,” and a “poetic feeling,” indicating that rhythmic patterning enhances affective involvement and aesthetic enjoyment.

**Table 7:** Number of Samples’ Comments on the Five Elements.

Translation strategies	Translations	Content	Theme	Affective involvement	Character Behavior	Typology	Number of readers
Prose translation	The Night Is Well On	42	16	25	37	60	119 (29.5%)
Free verse translation	The Jewel Stairs’ Grievance	20	21	17	17	35	63 (15.6%)
Rhymed translation	Waiting in Vain on Marble Steps	72	27	34	21	105	181 (44.9%)
Word-for-word translation	Yù jiē yuàn Yü chieh yüan Jade Steps Grievance	10	7	3	3	17	29 (7.2%)
Sum		145	67	79	78	217	392 (97.3%)

Specifically, those who favored Xu’s rhymed translation consistently emphasized its balanced integration of sense, form, and sound. In terms of content, samples noted that the translation “keeps the original meaning and poetic form while introducing rhymes that help its flow,” suggesting that semantic fidelity is reinforced rather than undermined by formal patterning. Many readers further observed that the rhymed version “maintains the tone and integrity of the original poem” and “expresses the gist and environment very clearly”.

With regard to thematic expression, samples perceived “a clear undertone” in the translation, in which “the themes of luxury and loneliness are easily seen.” This clarity was frequently associated with affective resonance: the rhymed version was described as one that “resonates most with readers,” “arouses feelings,” and “stimulates aesthetic pleasure and reflection.” Such responses indicate that rhyme functions not merely as a decorative device, but as a catalyst for emotional engagement and interpretive depth.

In terms of character behavior, readers found that Xu’s version “best establishes that the concubine was waiting outside for some time and continues to wait once the night forces her back indoors,” suggesting that rhythmic coherence helps sustain narrative continuity and reader immersion. Overall, samples repeatedly characterized the rhymed translation as “elegant,” “rhythmic,” “catchy,” “memorable,” and “the most poetic of all four versions.”

Importantly, several readers explicitly linked their preference for the rhymed version to target-language poetic conventions. Within the Anglo-American poetic tradition, rhyme and meter serve as salient markers of poeticity, shaping readers’ expectations of what constitutes a poem. As one respondent noted, “The rhyming nature of the translation better relates to Western audiences,” while another commented, “It suits my tastes as an English reader. It has rhyme and other conventions of English poetry, which makes it enjoyable.” One reader further explained that “the rhyming poem conveys the meaning while still being able to link words together to rhyme,” highlighting the successful integration of semantic transmission and formal pleasure.

In a word, this findings can be interpreted in light of Xu Yuanchong’s theory of literary translation, particularly beauty in sound, which emphasizes rhythm and rhyme as essential carriers of poetic pleasure. They suggest that rhyme facilitates affective involvement, memorability, and aesthetic recognition by aligning the translated poem with readers’ expectations of English poetic form. In Xu Yuanchong’s terms, this convergence reflects the realization of beauty in sound and the teleological goal of delectability, achieved through equalization rather than mechanical imitation of source prosody. For Western readers, whose poetic traditions—from ballads to sonnets—have long privileged metrical regularity and end rhyme, rhythmic coherence functions as a familiar aesthetic cue, facilitating emotional engagement and literary recognition.The qualitative data thus substantiate the statistical findings and demonstrate how translation strategy mediates reader reception at both cognitive and aesthetic levels.

### 4.3. Textual analysis of four translations

To illustrate better how the four translation strategies convey “three beauties”, i.e., the beauty in sound, sense, and form of Li Bai’s poem of “Yu Jie Yuan”, textual analyses on four versions are made for comparisons. Table 8 indicates Li Bai’s original poem and its four translation strategies in version 1, 2, 3 and 4 respectively.

**Table 8:** Li Bai’s Poem “Yu Jie Yuan” and Its Four Translations Strategies.

Original Poem		Prose translation form (version 1)	Free verse translation form (version 2)	Rhymed translation form (version 3)	Words for word translation form (version 4)
		Weng Xianliang’s version	Ezra Pound’s version	Xu Yuanchong’s version	The researcher’s version
Title	玉阶怨	The Night Is Well On	The Jewel Stairs’ Grievance	Waiting in Vain on Marble Steps	Yù jiē yuàn Yü chieh yüan Jade Steps Grievance
Line 1	玉阶生白露,	The night is well on; silken stockings can hardly keep out the cold rising from marble steps bedewed	The jeweled steps are already quite white with dew,	The marble steps with dew turn cold,	Yù jiē shēng bái lù Yü chieh sheng pai lu Jade steps generate white dew
Line 2	夜久侵罗袜。		It is so late that the dew soaks my gauze stocking,	Silk soles are wet when night grows old.	Yè jiǔ qīn luó wà yeh chiu ch’in lo wa Night long invade silk stockings
Line 3	却下水晶帘,	Retreating, she lets down the crystal curtains. Yet lingering, she turns her gaze upon the clear autumn moon.	And I let down the crystal curtain	She comes in, lowers crystal screen,	Què xià shuǐ jīng lián ch’üeh hsia shui ching lien But lower water cystal curtain
Line 4	玲珑望秋月。		And watch the moon through the clear autumn.	Still gazing at the moon serene.	Líng lóng wàng qiū yuè ling lung wang ch’iu yüeh Delicate subtle watch autumn moon

In Weng Xianling’s prose translation, “generalization” technique is adopted in translating the title and first two poetic lines. Weng makes a creative change and rephrased the title of the original poem into a sentence—“The Night Is Well On”, which brings out to readers the lonely and cold atmosphere on a dark and windy night. Weng further reinforces this bleak atmosphere by combining the first two poetic lines into one, repeating the title creatively and deleting the images “jade steps” and “white dew”. To highlight the theme and artistic conception of the poetry, Weng adopts “particularization” technique and strengthens animism in his translation, that is endowing plants, animals and natural phenomena with human attributes and characteristics in poetry. For example, in the second poetic line, silken stockings are given human traits that can prevent the cold, thus reinforcing the aesthetic impact of coldness and making the translation more intriguing.

In translating the third and fourth poetic lines, Weng Xianliang adopts “particularization” technique by adding “retreating” and “lingering”, which vividly present the protagonist (concubine)’s helplessness and disappointment due to her beloved (emperor)’s delay in seeing her. According to Weng [12], slightly providing guidance within the translation far surpasses detailed annotations outside the translation. By dint of deliberately transforming the “implicit” images of original poems into “explicit” meanings, the ideorealm of the original poems can be achieved. Furthermore, although Weng’s prose translation does not rhyme, his translation still conveys the musical effect and inner rhythm. For example, “retreating” and “lingering” have end rhymes, and “let down” and “turn upon” form antithesis.

**Fig 1:**
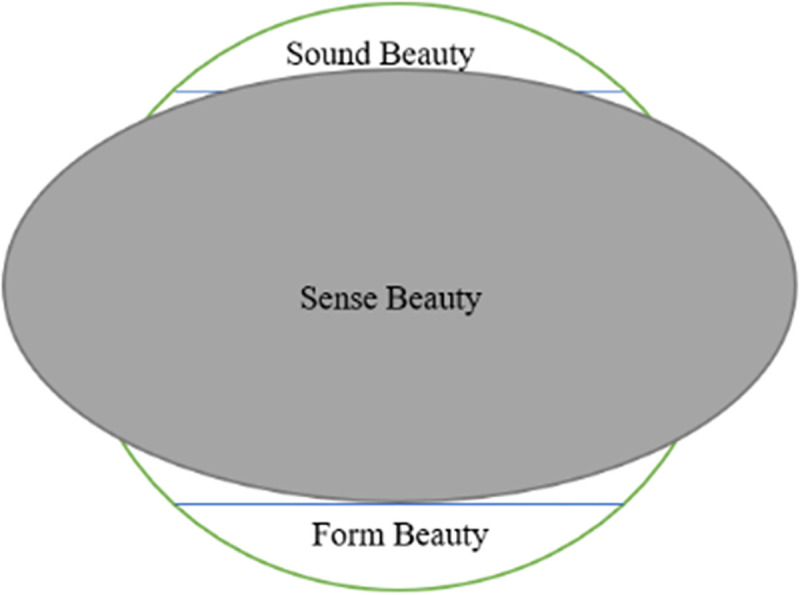
Weng’s Prose Translation Conveying the Beauty in Sound, Sense and Form of “Yu Jie Yuan”.

Thus, it can be seen that, by getting rid of the formal constraints of the poem, it is indeed more conducive for Weng to reconstruct the poems to highlight the artistic conception of the original poem through relevant techniques. However, his over-playing of creativity in translation by deleting certain important images for reconstruction of original poems can make the poetic lines unbalanced in form—the original four-line quatrain is converted into three lines with varying lengths. The beauty in sound, form and sense of the original poem conveyed in Weng Xianliang’s prose translation can be manifested in Fig 1.

As is shown from Fig 1, the green circle is the original poem with the beauty in sense, sound, and form; the grey oval represents Weng’s prose translation. In Weng Xianliang’s prose translation, the beauty in form is totally lost; the sound of the original poem is partially lost while the sense embodied in poetic ambience is too much amplified.

Ezra Pound is a leading Imagist poet adopting free verse. In translating the title “玉(Yù, jade) 阶 (jiē, steps) 怨(yuàn, grievance)”, Pound adopts “equalization” technique by converting the original “玉(Yù, jade)” into “Jewel”. “玉(Yù, jade) 阶 (jiē, steps)” is a laudatory name for steps made of marble, alluding to the high status of the protagonist living in the palace. For thousands of years, jade, considered as a sacred stone, is held in high regard. It can be associated with Confucian virtues such as wisdom, justice, purity and moral integrity. The highest echelons of Chinese society, including emperors and nobles, have been linked to jade since exquisite jewelry, decorations, and ceremonial artifacts frequently made with it can reflected their high social standing and grandeur. Pound’s transforming of the original cultural image of “jade” into “jewel” leads to the similar effect in Western context, indicating the high social status of the character, albeit with slight loss of other implications that “jade” could convey in Chinese context.

The original poem has two settings, outdoors and indoors. The first two lines describe how on a cold autumn night, the protagonist is waiting in vain on outdoor steps for her beloved to come to stay with her for the night and is unaware of the heavy dew soaking her stockings. Therefore, Pound’s title translation “jade staircase” does not conform to the outdoor setting of the first lines in the poem since “staircase” mostly refers to a set of stairs inside a building. Thus, Pound’s diction of “stairs” in the title is inappropriate.

In Pound’s translating of the body parts, he adopts a lot of “particularization” techniques by adding the logical connectives, making the version read more like a prose poem with line breaks. For example, “…are already quite white” is added in the first poetic line; “It is… that the dew” is added in the second poetic line; “and”, “I” are added in the third line; “and”, “through” are added in the last line. Since Pound does not know Chinese, his translation is mostly influenced by the notes made by the American Orientalist Fenollosa, who translates the cultural images “玉阶”, “白露”, “罗袜”, “水晶帘”, “玲珑”, “秋月” into “jewel stairs”, “white dew”, “gauze stocking”, “crystal curtain”, “transparent clear” and “autumn moon” respectively, which are almost preserved in Pound’s translation of the poem. However, the rendering of such images does not deeply convey the poetic ambience and protagonist’s sentiments in the translation. Due to the cultural differences between China and the West, Pound’s translation tends to remain at the level of surface equivalence of cultural images, lacking the exploration of their richer connotations.

**Fig 2:**
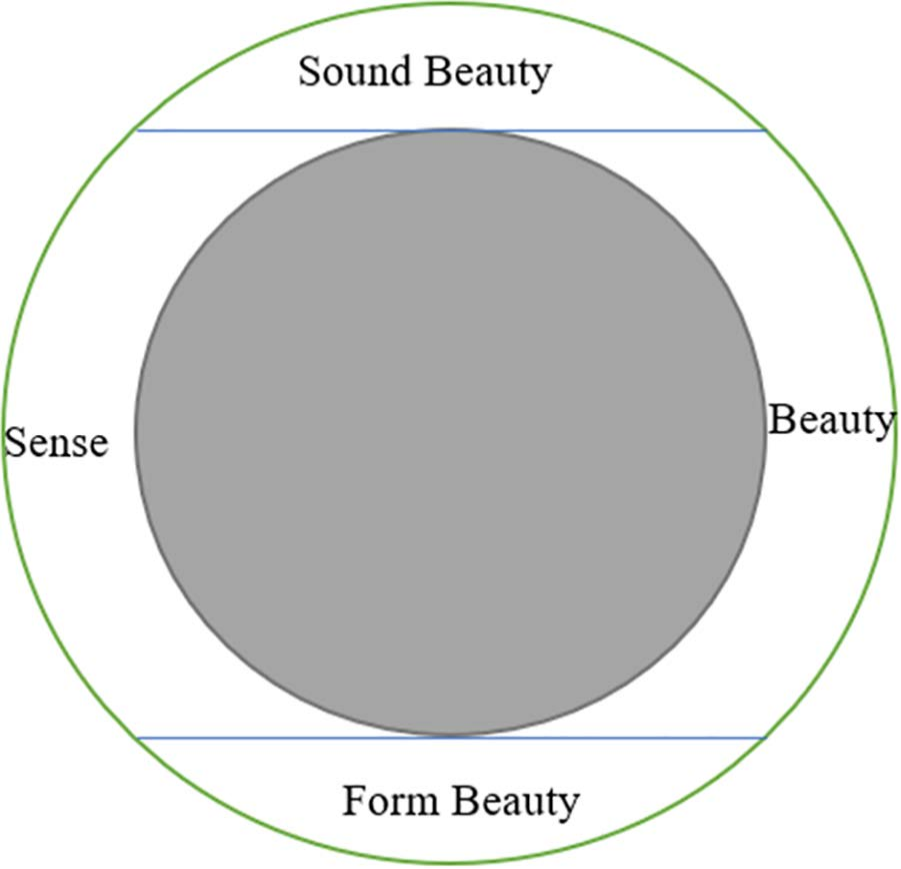
Pound’s Free Verse Translation Conveying the Beauty in Sound, Sense and Form of “Yu Jie Yuan”.

For example, in the last poetic line “玲珑望秋月”, “玲珑” (líng lóng) literally means something delicate or subtle. In the poem, it is a pun, not only indicating the delicate ringing sound of the “水晶帘” (shuǐ jīng lián), i.e., crystal curtain, but also the sereneness and subtleness of autumn moon. The poet Li Bai uses the cultural image of “玲珑” (líng lóng) to indicate to readers how the outdoor coldness drives her indoors, making her lowering the crystal curtains (thus making sound) and stares at the serene moon helplessly (thus setting off the character’s infinite disillusionment and grievances). Moon in Chinese context is mostly associated with reunion or nostalgia and used to express homesickness or lovesickness. However, Pound rendered the last line into “…and watch the moon through the clear autumn”, which did not convey the implicit beauty and nuanced cultural connotations of the pun “玲珑” (líng lóng), nor did it render the melancholy lovesickness of the character. Since moon in Western context is mostly symbolic of beauty, brightness, dreams or mystery, his translation gave readers a fresh feeling with a streak of lightness. Li Bai’s poetic ingenuity of “employing imageries to express emotions” is to some degree lost in Pound’s translation. In addition, the length of the lines in this translation varies, and there is no rhythmic pattern to speak of. The beauty in sound and form are therefore lost as well. The beauty in sound, form and sense of the original poem conveyed in Pound’s free verse translation can be manifested in Fig 2.

In Fig 2, the green circle represents the original poem with the beauty in sense, sound, and form; the grey oval represents Pound’s free verse translation. In Pound’s free verse translation strategy, the beauty in form and sound of the original poem are totally lost while the sense embodied in poetic ambience is reduced by his surface meaning rendering of original cultural images. As Xu Yuanchong [42] maintained, the free verse poets of the Imagist school, focusing only on the imagery instead of ideorealm, theoretically could not translate classical Chinese poems featured by fewer characters with more profound meanings.

In rhymed translation, Xu adopts “generalization” technique in translating the title “玉(Yù, jade) 阶(jiē, steps)怨(yuàn, grievance)” by omitting the word “怨(yuàn, grievance)” and simplifying it into “waiting in vain”, explicitly expressing the protagonist’s deep resentment and disappointment. Besides, Xu deliberately adopts dactylic meter in the title, where the rhythm of dropping from the stressed syllables to the unstressed syllables creates the verse with great rhythmic dash and generates a sense of loneliness and sadness, thus successfully setting the melancholy tone for the poem from the title.

In the first poetic line, “玉阶生白露 (Yù jiē shēng bái lù, literally meaning that jade steps generate white dew)”, the original poet Li Bai uses such concrete images as “jade steps” and “white dew” to render the unstated gloominess of coldness of the poetic ambience. White Dew (bái lù白露) is 15th solar term of the year in China, indicating the real beginning of cool autumn. From the first day of the White Dew on, the temperature declines gradually and the vapors in the air often condense into white dew on the grass and trees at night. Xu Yuanchong adopts “particularization” technique by describing dew-soaked jade steps “turning cold” to render the coldness of the night and the intensity of the grievances, conveying the sense beauty of the original poem. Through interpretating the implied and subtle meaning of intriguing imageries—“jade steps” and “white dew”, Xu helps Western readers to perceive and appreciate better the beauty of poem. In the last poetic line, “particularization” technique is adopted by adding the words “still” and “serene” to end-rhyme the last word “screen” in the third poetic line, leaving much imagination for readers about the concubine’s melancholy of gazing at the moon through the curtain while waiting for the emperor, which manifests the traditional poetic tendency of “境生象外(jìng shēng xiàng wài)”, that is to say, the aesthetic conception evoked by a poem transcends what the image of concrete object denotes.

In CCP, the metrical pattern is reflected by the level and oblique tones, i.e., 平(píng) and 仄(zè), according to which, the metrical pattern of “玉 (Yù, jade) 阶 (jiē, steps) 怨 (yuàn, grievance)” is “仄平平仄仄, 仄 仄平平仄. 仄仄仄平平, 平平仄平仄”. Since the original poem is a five-character jueju, that is a pentasyllabic quatrain, the rhythmic stress normally falls on the second, fourth and fifth character of the line—“阶jiē”, “白bái”, “露lù”, “久jiǔ”, “罗luó”,“袜wà”,“下xià”,“晶jīng,”, “帘lián”, “珑lóng”, “秋qiū”, “月yuè”. Xu’s translation adopts an iambic tetrameter with an a-a-b-b rhyme scheme, corresponding to the rhythmic effect of Chinese metrical pattern of the poem, thus conveying the beauty in sound.

**Fig 3:**
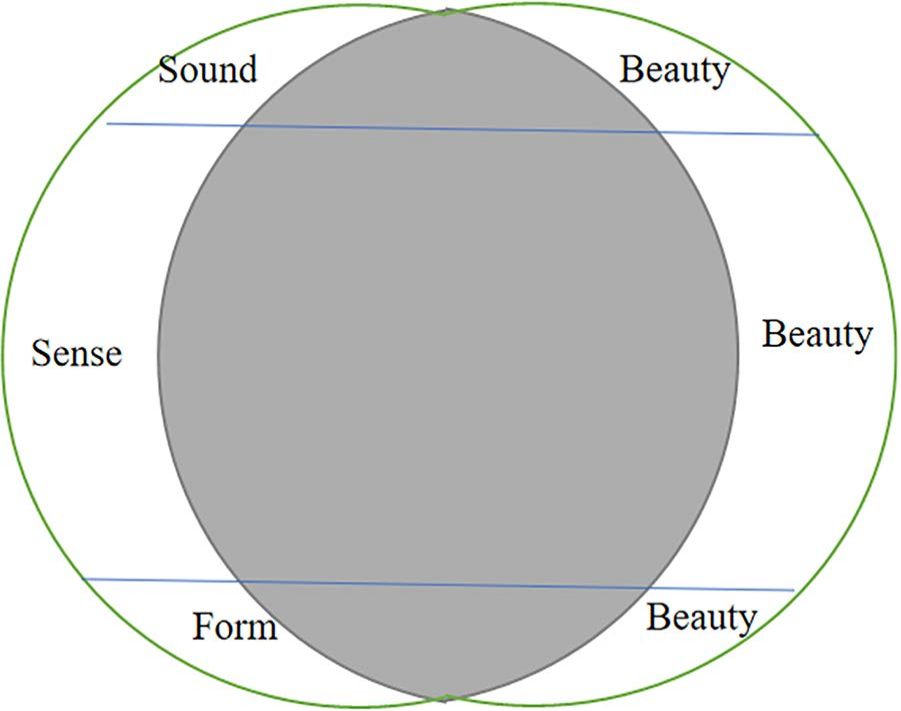
Xu Yuanchong’s Rhymed Translation Conveying the Beauty in Sound, Sense and Form of “Yu Jie Yuan”.

In terms of the poetic form, the Chinese character “生” (shēng, meaning “generate”) in the first poetic line is paired with the character “侵” (qīn, meaning “invade”) in the second poetic line. Correspondingly, in Xu’s translation, the words “turn” and “grow” are paired with each other in a rigorous and neat manner, reproducing the form beauty of the original poem. The three aspects of beauties of original poems reflected in Xu’s translation can be illustrated in Fig 3.

As is shown from Fig 3., the left circle is the original poem with the beauty in sense, sound, and form; the right circle represents Xu Yuanchong’s rhymed translation, and the two overlapping gray ovals are the part of his rhymed translation that can convey the three beauties of the original poem. The grey crescent on the left represents the part that cannot be conveyed, and the grey crescent on the right represents the new “three beauties” created by the translator. By dint of maximizing advantages of target language, Xu Yuanchong flexibly conveyed three aspects of beauty of CCP in his translation, making target readers understandable, enjoyable and detectable, which has been favored by most readers.

**Fig 4:**
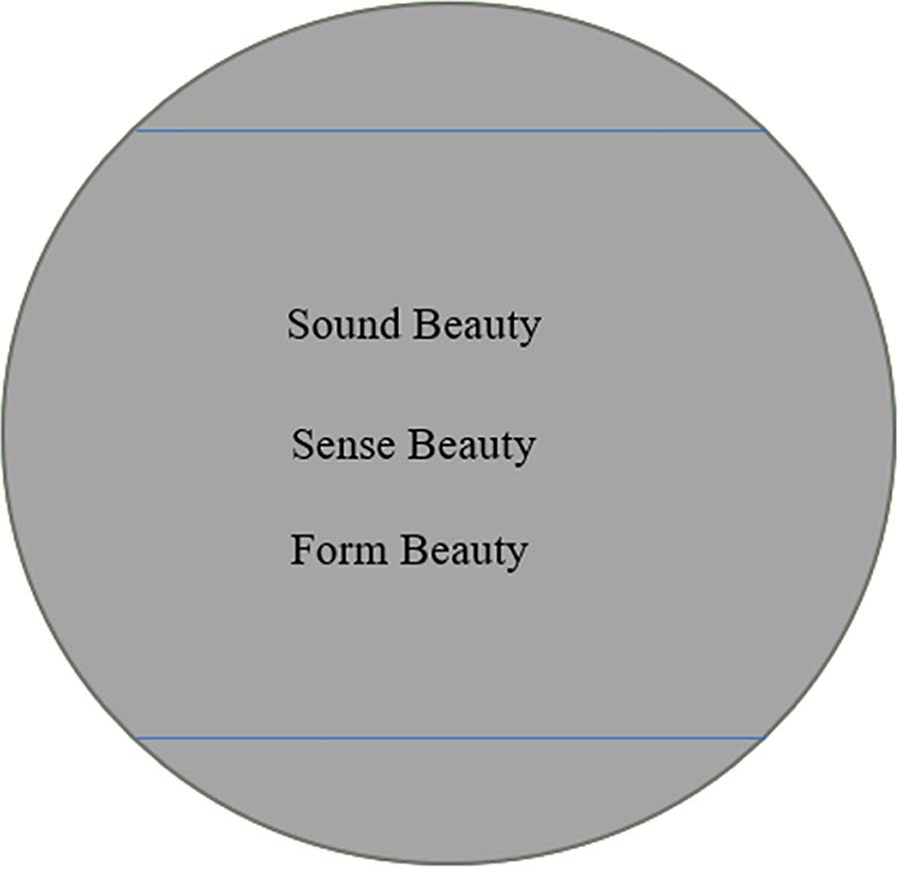
Word-for-Word Translation Conveying CCP’s Beauty in Sound, Sense and Form of “Yu Jie Yuan”.

In the book West-östlicher Divan, Goethe illustrates three epochs of translations. The first epoch “familiarizes us with the foreign country on our own terms” [41]. It is like letting the foreigner put on our clothes and appear in our familiar appearance, which looks more amicable and less strange. In the second epoch, the translator endeavors to enter the foreign situation, but what the translator does is to “appropriate the foreign meaning and then replaces it with one’s own” [52] so that the translation can surpass the original text and elongate the life of the latter. Goethe calls it the parodistic translation through which foreign fruits can be transplanted into a surrogate grown in their own soil. In the third epoch, the translator seeks to make the translation identical with the original, being a true replacement for the original instead of a substitute. This stage is, according to Goethe, “the last and highest of all” by Goethe — “A translation that seeks to be identified with the original approximates, finally, the interlinear version; in its attempt to enhance our understanding of the original it leads us onward, drives us on toward the source text, and so finally closes the circle in which the alien and the familiar, the known and the unknown move toward each other” [52]. Goethe’s third translation epoch is characterized by the word-for-word translation, which is an interlinear version that strives for consistency with the original in an ideal manner. It deepens our understanding of the original. At this point, the circles representing the original poem and the target translation can finally merge together as can be shown from Fig 4.

It is worth noting that, Goethe does not rigidly adhere to a linear development of the three translation epochs. As Goethe indicates, these three epochs can be “repeated and reversed” in every literature, and the strategies they represent can be employed simultaneously [52]. In fact, the strategies used in three translation epochs can co-exist and complement each other, each playing its own unique role. Samples’ less satisfaction with the word-for-word translation strategy reflects the difficult operationalization of putting it into practice since it is “disjointed” and sometimes “doesn’t quite make sense in English” as one sample in RRQ commented. The ideal solution is to combine word-for-word translation with other translations so that target readers could gain access to the original poem and have a better understanding of the sense beauty, form beauty and sound beauty of CCP.

## 5. Discussions

Through an empirical investigation combining quantitative ratings, qualitative reader responses and textual analysis of four translations, this study demonstrates that rhymed translation strategy serves as a better option in rendering CCP. The finding highlights how poetic form operates as a site of cross-cultural negotiation between source-text aesthetics and target-culture expectations.

Rather than treating rhyme as a self-evident aesthetic advantage, the findings suggest that readers’ preferences reflect culturally conditioned notions of poeticity embedded in English literary traditions. For many participants, rhyme functions as a cognitive and affective marker that signals “poetry” as distinct from prose or everyday language. In this sense, the attraction to rhymed translation is not reducible to sound pleasure alone, but points to a deeper expectation that poetry should exhibit patterned musicality and formal closure. This observation complicates the widespread assumption that modern Anglophone readers favor free verse and challenges the view that rhyme is necessarily perceived as artificial or outdated in English poetry.

From the perspective of Xu Yuanchong’s Three Beauties Theory, the empirical data highlight the centrality of beauty in sound in reader reception. However, the findings also suggest that sound cannot be isolated from sense and form. Qualitative responses indicate that readers value rhyme most when it enhances clarity, memorability, and emotional resonance, but reacts negatively when formal constraints interfered with comprehension. This conditional appreciation underscores Xu’s notion of equalization, in which aesthetic effects are recreated through negotiation rather than mechanical imitation. In this respect, the study reinforces Xu’s theoretical claims by grounding them in reader-based evidence: beauty in sound is effective only insofar as it remains intelligible and semantically anchored. It also challenges rigid form-oriented or meaning-oriented positions and supports a relational view of fidelity, in which ethical translation practice responds to reader cognition and cultural norms without erasing source-text aesthetics.

From a cross-cultural perspective, the positive reception of rhymed translation can be further understood through the functional correspondence between Chinese and English prosodic systems. Although CCP relies on tonal regulation while English poetry is stress-based, both traditions employ patterned sound to organize emotion and meaning. Xu Yuanchong’s use of iambic meter and end rhyme exemplifies how functional equivalence can be achieved across linguistic systems, enabling Western readers to experience a form of poetic discipline analogous to that of regulated Chinese verse. In this way, rhyme does not impose a foreign structure, but rather serves as a mediating device that facilitates cross-cultural poetic understanding.

It should be noted that different translation strategies—rhymed, free verse, prose, and word-for-word—should be understood relationally rather than hierarchically. The empirical preference for rhymed translation observed in the present study does not imply the categorical superiority of rhyme over other strategies, but indicates its relative effectiveness under specific conditions, namely the translation of classical Chinese poetry for contemporary English-speaking readers. As Goethe observes, translation strategies do not follow a linear historical progression but coexist and recur within literary systems, each fulfilling distinct communicative and aesthetic functions. It adds strong support to Ma’s [27] claim that “Any single translation form has its own insurmountable defects…Rhymed translation, free verse translation, prose translation and word-for-word translation should be combined altogether with the para-text to form a complete translation product”. This reinforces the view that poetry translation is best conceived as a plural and dialogic practice, in which different strategies achieve relative optimality under different conditions, rather than as a search for a single universally optimal form.

## 6. Conclusion

This study investigates target readers’ reception of different translation strategies in English versions of Li Bai’s “Yu Jie Yuan” through an empirical mixed-methods approach. It is found that rhyme functions as a culturally salient indicator of poeticity for American readers, enhancing aesthetic engagement when semantic clarity is maintained. Rather than treating rhyme as an inherently superior strategy, the results demonstrate that the effectiveness of rhyme thus lies not in formal replication per se, but in its functional coordination with semantic intelligibility and tonal coherence. This insight contributes to translation studies by empirically supporting a non-hierarchical view of translation strategies, in which rhyme, free verse, prose, and literal rendering function as context-sensitive options rather than competing ideals.

At a theoretical level, the study bridges reader reception theory and poetic translation research by showing how aesthetic value in translation emerges through negotiation between source-text features and target readers’ expectations. In doing so, it offers reader-based empirical support for Xu Yuanchong’s aesthetic principles, while simultaneously situating them within a broader intercultural framework that emphasizes dialogism, variability, and audience orientation. Poetry translation is neither a unidirectional transfer of form nor a purely subjective recreation, but a dynamic site where cultural norms of poetic appreciation are activated, challenged, and reconfigured.

More broadly, the study underscores the role of translation in intercultural literary communication. The reception of CCP in English translation is revealed as an active process of aesthetic sense-making, in which readers draw upon familiar poetic markers—such as rhyme—to access an unfamiliar literary tradition. When guided by functional equivalence rather than mechanical imitation, rhymed translation may serve as a mediating device that enhances cross-cultural poetic appreciation, without foreclosing alternative translational possibilities.

Several limitations should be acknowledged. The participant pool was restricted to American readers, which limits the cross-cultural generalizability of the findings. Future research could incorporate more diverse readerships, compare responses across cultural and linguistic backgrounds, and employ additional qualitative methods such as focus group discussions or longitudinal reception studies. Such extensions would further clarify how translation strategies interact with culturally specific notions of poetry and deepen our understanding of poetic reception in a global context.

## Supporting information

S1 File Anonymized dataset from the questionnaire survey. This file contains the anonymized raw data collected for the study, including demographic variables and responses to all questionnaire items. The dataset serves as the basis for the statistical analyses reported in the article.
(XLSX)
